# Neurobiochemical and psychological factors influencing the eating behaviors and attitudes in anorexia nervosa

**DOI:** 10.1007/s13105-016-0540-2

**Published:** 2016-12-06

**Authors:** Teresa Grzelak, Agata Dutkiewicz, Elzbieta Paszynska, Monika Dmitrzak-Weglarz, Agnieszka Slopien, Marta Tyszkiewicz-Nwafor

**Affiliations:** 1 Division of Biology of Civilization-Linked Diseases, Department of Chemistry and Clinical Biochemistry, Poznan University of Medical Sciences, Swiecickiego Str. 6, 60-781 Poznan, Poland; 2 Department of Child and Adolescent Psychiatry, Poznan University of Medical Sciences, Poznan, Poland; 3 Department of Biomaterials and Experimental Dentistry, Poznan University of Medical Sciences, Poznan, Poland; 4 Psychiatric Genetics Unit, Department of Psychiatry, Poznan University of Medical Sciences, Poznan, Poland

**Keywords:** Anorexia nervosa, Eating disorders, Nutritional attitudes, Nutritional behaviors

## Abstract

The aim of this study was to determine the characteristic features which contribute to inappropriate eating attitudes in people suffering from anorexia nervosa, based on an analysis of recent data. Factors influencing these attitudes have a genetic, neurobiological, biochemical, affective-motivational, cognitive, and behavioral background. Another important issue addressed in the paper is a description of the mechanism leading to continuous dietary restrictions. The altered activity of neurotransmitters modulating patients’ moods after the consumption of food and a disturbed responsiveness to enterohormones enhance affective-motivational and cognitive aspects which, in turn, impede the improvement of eating behaviors. An understanding of the mechanisms behind the factors affecting the maintenance of inappropriate eating attitudes may contribute to greater effectiveness in the treatment of anorexia nervosa.

## Introduction

**Fig. 1:**
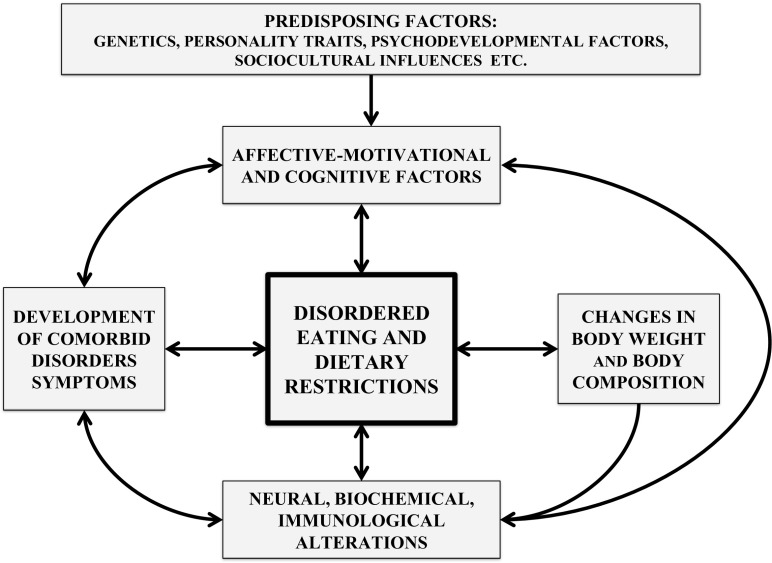
Factors supporting the restricted eating of patients with AN

Anorexia nervosa (AN) is a multietiological disorder predominantly affecting adolescent girls (Fig. 1). It is characterized by a change in eating behavior that leads to a reduced and maintained low body weight below the recommended level for age and gender. The disorder is accompanied by numerous psychological symptoms such as intense fear of gaining weight, obsessive-compulsive behaviors or affective disorders including depression. The social functioning of people suffering from this condition also changes. They tend to isolate themselves, distrust their surroundings, are unwilling to conform to social rules and norms and often display a sense of moral superiority [15, 21, 49]. Patients become severely malnourished and significantly emaciated, leading to endocrinological and cardiological dysfunctions as well as to numerous abnormalities within the digestive, skeletal, and reproductive systems [50, 51]. These dysfunctions co-occur with biochemical, hematological, and dermatological changes. Somatic complications are often life-threatening for patients suffering from AN. The important symptoms of AN include distinctive food-related attitudes and behaviors which determine the patients’ diet. Reduced food intake is the main manifestation of this disorder. However, the refusal to eat has an individualized level of strictness. This diversity depends on the subtype of AN (restrictive or bulimic—purging), the course and stage of the disorder, as well as the range of individual factors. AN sufferers are intuitively perceived by society as people who submit themselves to chronic self-imposed starvation [50]. The factors underpinning anorexic patients’ abnormal dietary attitudes have various underlying causes. The aim of this paper is to characterize the neurobiological, behavioral, emotional, and cognitive factors determining these attitudes as well as the mechanisms conducive to the dietary restrictions of people suffering from AN and is based on the current data in the literature.

## Genetic and neurobiochemical factors

**Fig. 2:**
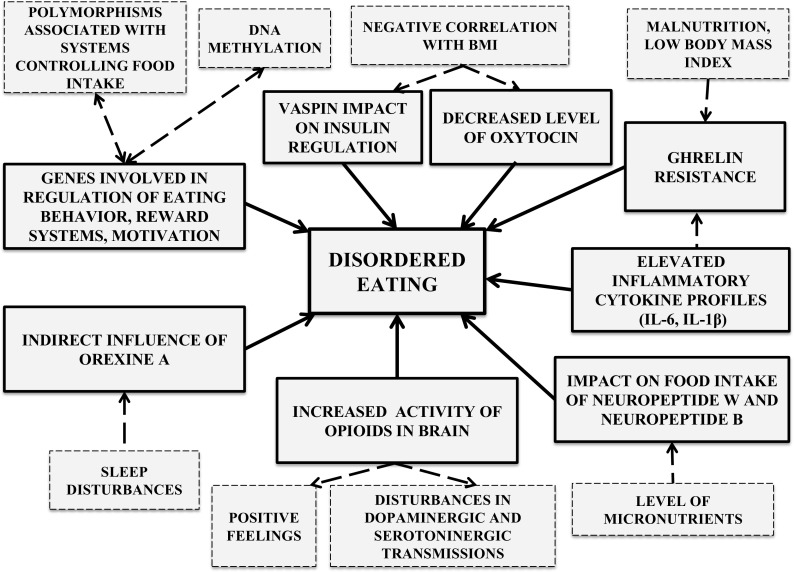
Biological factors supporting the restricted eating habits of patients with AN

A growing body of family, twin and adoption studies has certified a link between AN and genetic factors. The current state of knowledge leads to the conclusion that AN is a heritable condition [37, 57]. Data from twin studies remains ambiguous because some sources report a heritability rate of AN of about 28% and others as high as 58% [57]. So far, nearly 130 polymorphisms have been detected on 43 genes, among which are the genes responsible for personality traits and tendencies in the way of emotion regulation, but also in genes involved in eating attitudes, the regulation of eating behavior, motivation, and reward mechanisms (Fig. 2) [4, 36]. Scientists have attached considerable importance to those genetic factors involved in the systems controlling food intake, such as, the polymorphisms associated with the agouti-related peptide, opioid receptor delta-1, or brain derived neurotrophic factor [36]. Researchers also emphasize the role of epigenetics with preliminary hypotheses assuming that DNA methylation may contribute to the development of AN [35].

Avoidance of meals accompanied by increased energy expenditure, principally due to intensive physical activity, is practiced by patients with AN in order to achieve and maintain an extremely low body mass and a thin appearance [60]. Prolonged reduction of nutrient intake by AN patients increases the activity of opioids in their brain, which trigger positive feelings and enables their adherence to a restrictive diet. Furthermore, food consumption reduces optimism and intensifies anxiety [25, 39]. PET (positron emission tomography) and SPECT (single-photon emission computed tomography) tests have found disturbances in dopaminergic and serotoninergic transmissions in AN. A strong binding between dopamine D2 and D3 receptors in anterior ventral striatum, as well as an intensified 5-HT1A and reduced 5-HT2A receptor binding are reported. These abnormalities are related to emotional reactions which, in turn, influence patients’ food-related behavior. This finding was confirmed in studies which proved that, in AN patients even after recovery, the release of endogenous dopamine from striatum was related to experiencing a state of increased anxiety, as opposed to a control group with no history of eating disorders who were in a better mood as a result of dopamine release from anterior ventral striatum. Normally, the consumption of a delicious meal causes dopamine release in the striatum but, in cases of endogenous dopamine release, people suffering from AN experience intensified anxiety instead of positive emotions. This may explain their desire to continue self-starvation and dietary restrictions [25]. Moreover, AN patients tested with the use of fMRI (functional magnetic resonance imaging) showed abnormalities within hunger and satiety centers. The processing of food-related somatosensory stimuli in the state of satiety was reduced, in comparison with healthy individuals, and the occurrence of mechanisms enabling dietary restrictions was observed [41].

Ghrelin resistance, which develops in the state of malnutrition, appears to be a conducive factor to a restrictive diet. Ghrelin is an enterohormone secreted in a pulsate manner, principally by the mucous membrane cells of the fundus of the stomach and other parts of the digestive system. This peptide significantly influences the energy homeostasis of the body. It activates NPY/AgRP (neuropeptide Y/agouti-related peptide) neurons as well as others involved in the production of orexins, which stimulate the intake of food. It has been shown that the ghrelin level correlates negatively with insulin and leptin levels, as well as with the body mass index [11, 58]. Patients with AN have raised blood levels of ghrelin, which may be a compensating mechanism. However, this does not produce the expected effect of increased food consumption. Additionally, disturbances in the secretion of this enterohormone after meal consumption by AN people can be observed. This supports the validity of the hypothesis concerning ghrelin resistance involvement in the pathogenic mechanism of the disorder. Ghrelin resistance may also be responsible for the fact that AN patients do not abstain from small food intake [34, 60].

It is known that the presence of inflammation may also affect the functioning of the neuroendocrine system and modulate the secretion of some of the substances mentioned above. It is known that interactions between the endocrine, nervous, and immune systems influence effective immune responses [7]. Long-term extreme calorie restriction and/or changes in body composition, especially the low body fat stores which are observed in patients suffering from AN, are correlated with some changes in the immune system. The results of a recent meta-analysis concerning the presence of inflammatory cytokines in AN [47] show that the condition is associated with significantly elevated levels of inflammatory cytokines, in comparison to healthy control subjects. In particular, there are significant differences in the levels of TNF-α, IL-6 and IL-1β. However, it is also observed that after AN patients gain weight, IL-6 is maintained at normal levels [47]. Studies of some animal models focusing on the relation of inflammatory cytokines to ghrelin suggest that the expression of ghrelin may be regulated by pro-inflammatory cytokines [28]. This could explain the indirect impact of inflammation on the eating behavior of AN individuals. Solmi et al. conclude their meta-analysis with the suggestion that future studies might explore whether inflammatory cytokines could become new treatment targets in AN [47].

Another example of substances which play an important role in food intake regulation are the orexins. Orexin-A (OXA) is a 33-amino acid orexigenic neuropeptide implicated in eating behavior regulation and is also responsible for the sleep-wake cycle [42]. Fasting or food intake restriction in healthy-weight humans results in a gradual increase in serum OXA, which normalizes with re-feeding. In AN patients, the changes in the concentration of OXA are ambiguous. Some studies indicate either increasing or decreasing plasma levels of OXA [5, 22]. This suggests that OXA concentrations do not appear to be linked to body composition (body fat store) or to the BMI, but the level of secretion may be regulated by energy status [5, 42]. Studies have indicated that the concentration of OXA in patients with AN does not affect the outcome directly, but does so indirectly, through the regulation of sleep and wakefulness [42].

In recent years, researchers have identified and described several new molecules that might be of importance for the diagnosis and the outcome of AN. These include neuropeptide W, neuropeptide B and vaspin, although their exact roles and contribution to this disorder are not fully known, since they mainly involve animal studies. Neuropeptide W is a recently discovered, endogenous ligand of GPR7 and GPR8 receptors, which belongs to the GPCR receptor family (G-protein-coupled receptors) and participates in energy homeostasis, regulation of the hypothalamic-pituitary-adrenal axis and the secretion of some hormones [54]. Relatively large amounts of mRNA of the neuropeptide W gene have been detected in the human brain, hippocampus, substantia nigra, stomach, and rectum [53]. Its expression in the stomach was found to depend on the level of nourishment and the type of food consumed [29]. Injections of high doses of neuropeptide W (3 nmoL/100 g of body mass) caused a short-term decrease in the leptin and insulin levels in rat blood. However, no changes were observed following the administration of neuropeptide B—a polypeptide of similar structure to neuropeptide W, which also appears to be an endogenous ligand of GPR7 and GPR8 receptors [38]. Intraventricular administration of small doses of neuropeptide B to mice induced hyperphagia and hyperactivity but in high doses—hypophagia and even AN [55]. No data concerning studies on neuropeptide W and B levels in individuals with AN was found.

It has been suggested that vaspin (serine protease inhibitor) may be an antagonist of some as yet unknown molecules which reduce the activity of insulin. Suwala et al. have shown that there are higher levels of this protein in the blood of AN patients, when compared to a group of people with a normal body mass [52]. Moreover, a negative correlation was found between the serum vaspin level and body mass in individuals with AN as well as in a control group without this disorder. Vaspin is produced in visceral and subcutaneous adipose tissue as well as in the cells of the hypothalamus, pancreas, stomach, and cerebrospinal fluid [30].

The concentration of oxytocin in the serum of people with AN was half that in an age-matched comparison group. In addition, the level of this hormone negatively correlated with the body fat mass, leptin level and bone mass density in AN patients [27]. Recent studies have shown a correlation between the BMI and the level of methylation oxytocin receptor gene in patients with anorexia. The level of epigenetic modification of this gene differed significantly in the groups with, and without, AN [26]. Recently, the results of studies which showed that nasally administered oxytocin may be useful in the treatment of anorexia have been published [26].

## Emotional factors

**Fig. 3:**
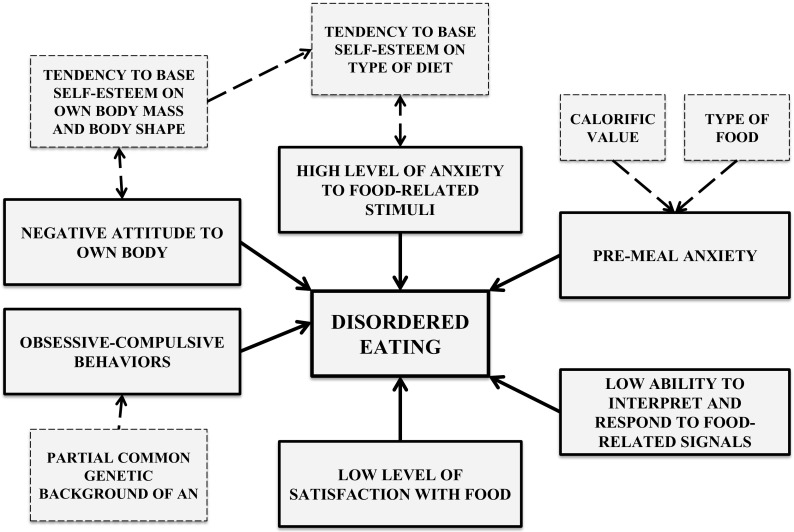
Emotional and obsessive-compulsive factors supporting the restricted eating habits of patients with AN

Every attitude and behavior in AN subjects has an affective and motivational component (Fig. 3). Their regulatory function consists of, among other things, generating the reinforcement of some actions taken. Negative emotions prompt an individual to act in such a manner that would reduce these actions, and this usually involves avoidance of the stimulus or halting the activity. Searching for possible emotional causes of dietary restrictions by AN patients therefore appears justified.

The study conducted by Steinglass et al. confirmed that the maintenance of dysfunctional eating behaviors by AN sufferers is largely related to pre-meal anxiety, the level of which is dependent on the calorific value of the meal [23, 49]. The hypothesis concerning an anhedonic attitude of patients towards food is a starting point for conducting a study regarding the level of satisfaction experienced during visual and olfactory exposure to food stimuli, depending on the calorific value of the stimulus and the state of the satiety of subject. It was found that AN sufferers have a selective reduction in the pleasure experienced, depending on the type of stimulus. If it was related to food, the subjects experienced a statistically significantly lower rate of satisfaction, in comparison to that of the controls. For example, the higher the calorific value of the food, the more intense feeling of satisfaction experienced by the healthy subjects than by the AN patients. Moreover, the authors of the study, following their analysis of the results of the desirability level, calculated with regard to a particular stimulus, noted that the level does not change in AN patients in contrast with the healthy subjects. This may indicate a lower ability of AN patients to interpret and respond to food-related signals [23].

A very strong motivational factor in human life is to prevent a reduction in one’s self- esteem. People suffering from AN tend to base their self-esteem on their appearance and body mass, which results in their pursuit of the extremely low anthropometric measurements which they consider ideal [9]. From this point of view, it might be argued that the maintenance of abnormal food-related attitudes and behaviors in patients with AN is, paradoxically, an adaptive mechanism which enables them to reduce negative emotions occurring when they are faced with a threat to their self-esteem.

## Cognitive factors

**Fig. 4:**
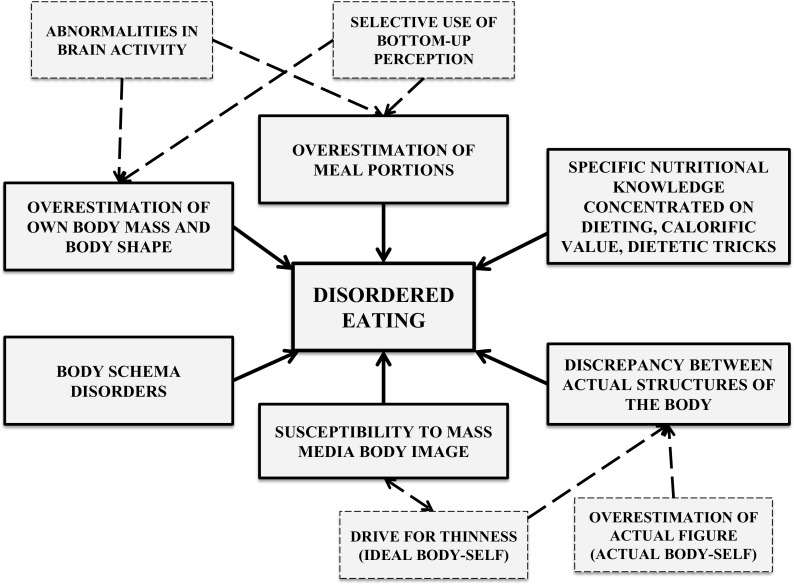
Cognitive factors supporting the restricted eating habits of patients with AN

A distinctive way of body self-perception, as well as a conversion of food-related stimuli, is often quoted as a characteristic feature of AN (Fig. 4) [20, 32, 46, 56, 59]. Body image, to which the structure of ego, “body-self” is related, is a construction determining body self-perception (“body perception”) and the attitude towards it (“body concept”). According to Higgins’ self-discrepancy theory, the messages incorporated into the structure of body ego influence the level of self-knowledge and self-esteem, with a distinction between the state perceived as actual (“actual self”), the state that is aspired to (“ideal self”), and the state desired by society (“ought self”). Studies have confirmed that individuals suffering from AN have discrepancies between these structures. Their source is, on the one hand, the perception that an emaciated frame is the proper one, and on the other—a false perception of their own appearance [56].

The incorrect evaluation of their shape and the overestimation of their own body measurements exhibited by individuals suffering from AN have attracted the interest of many researchers [20, 46]. Hamilton and Waller, while searching for the roots of body schema disorders, proved that women with eating disorders are more susceptible to the thin body ideal shown in the media in evaluating the size of their own bodies. Even a few-minute-exposure to photos showing such body images promoted by the media caused statistically significant rises in the overestimation of body self-perception [20]. The study of Smeets et al. focusing on body size perception attempted to determine the underlying causes of disorders in the perception of body characteristics. The authors of this study suggested that it must not be assumed that disorders related to body scheme estimation are underlain by a disturbance in the perception process, understood as the reception of visual data. This process they termed “bottom-up”. Instead, they indicated a potential source of the problem in perception, based on an initial interpretation of the data (predetermined by the subjects’ knowledge, expectations, and context). This organizes the mode of stimuli perception—the “top-down” process (Fig. 4). This hypothesis is supported by the fact that, while retrieving the appearance of the body from memory, the body image disturbances were more pronounced than during a visual evaluation [46]. Furthermore, there is also a neural basis for the disturbances in self-perception in AN and some studies have confirmed its clinical relevance [8, 16]. Researchers, using the fMRI method to examine body image processing, found different patterns of neural response mainly involving the superior and inferior parietal lobules and the dorsolateral prefrontal cortex [8]. Gaudio and Quattrocchi, in their study involving a multidimensional model of body image distortion in AN, revealed that specific neural alterations are related to the various components of the body image attitude [17]. The authors noticed that the perspective component is associated with alterations in the inferior parietal lobe and the precuneus, while the emotional aspect is mainly related to alterations in the amygdala, prefrontal cortex, and insula [17]. These findings show that self-perception in AN is a very complex issue. This interpretation, based on abnormalities in neural response, combined with a cognitive exploration of discrepancies between the actual self and the ideal self, intensify the negative emotions that an individual tries to reduce by approaching the ideal state which, in the case of people with AN, is achieved by applying dietary restrictions and other techniques aimed at body mass control.

Another factor which can potentially induce a smaller intake of food is a different estimation of meal portion sizes by AN patients. Visual evaluation of food amount has been the subject of many studies. Initially, the results unambiguously showed a tendency by AN individuals to overestimate meal portion sizes [30]. Subsequent analyses of stimuli, carried out by Vinai et al., based on estimations of the amount of similar portions, including edible (yellow sweets) and non- edible (yellow Lego pieces), surprisingly did not show statistically significant differences between the test and the control groups. One interpretation of these results was a hypothesis that people affected by AN perceive the amount in an objectively correct way but, with regard to their own ability to consume food, interpret the meal portion as too large [59]. Further attempts to verify this hypothesis have led to more definite conclusions. The study conducted by Milos et al., published in 2013, showed that statistically significant overestimation of portion sizes occurred when subjects were asked to imagine themselves having to eat the presented meal. Interestingly, AN patients tended to overestimate small or standard size portions but, in the case of above- average portions, this tendency was not observed. This information is crucial to understanding certain compensating behaviors as well as to the planning of nutritional therapy for individuals with anorexia. Swiss researchers also noted that AN subjects more often overestimated meal portion sizes when they were hungry, as compared to individuals at a more advanced stage of AN, who made greater errors in assessment (perceiving the portions as larger) [32]. As in the case of body image estimation, the perception of food stimuli in AN is very complex and is reflected in neural responses. fMRI studies on patients with AN detected the presence of differences in activation in some brain areas while thinking about eating the food shown in images [6]. During the experiment Brooks et al. noticed an increase in visual and prefrontal cortical neural responses in AN patients which may underlie their thinking about dietary restrictions [6]. Another fMRI study revealed dysfunctional activation of the brain areas involved in taste perception in AN individuals. Dysregulation of the brain reward mechanism is also observed, despite the character of the stimulus applied—abnormalities in brain activity following exposure to pleasant or aversive food stimuli [6, 33].

The population of eating disorder sufferers differs from healthy people with regard to their nutritional knowledge [12, 44]. Studies have confirmed the thesis that patients diagnosed with AN possess a greater knowledge of nutritional information than other social groups [12, 44]. At the same time it should be emphasized that the nutritional knowledge of these individuals is selective. It often focuses only on weight loss and the calorific value of food products and some of the information remembered is inaccurate and distorted [2, 12]. Despite their considerable interest in dietetics, demonstrated by their magazine and online searches as well as by their looking for information in popular science books, people with AN display statistically significant lower levels of nutritional knowledge in comparison to those of food science students [43]. Thus, the information gathered regarding nutrition has an influence on the dietary behaviors of AN subjects and the formation of their specific eating habits.

## Obsessive-compulsive predisposition

The relationship between AN and obsessive-compulsive symptoms has been widely discussed in the literature [1, 18, 24, 40, 48]. Obsessive-compulsive disorder is a type of anxiety characterized by experiencing intrusive thoughts (obsessions) and/or the performance of compulsory actions (compulsions). Researchers generally describe the relationship between these disorders as a comorbidity. However, in the literature, there are attempts to determine one of the diseases as a consequence of the other [1, 24, 40, 48]. Efforts have been made to incorporate AN into the obsessive-compulsive spectrum and to consider it as a specific subtype of obsessive-compulsive disorder, supporting this approach with claims of a common serotoninergic etiology [24, 45]. There is also the hypothesis of a partial common genetic background of both AN and obsessive-compulsive disorders. Mas et al., in their association study, revealed some interactions among a number of significant single-nucleotide polymorphisms (rs1074815 (*TPH2*); rs11783752 (*SCL18A1*); rs10070190 (*CDH9*); rs2834070 (*OLIG2*); rs3825885 (*NTRK3);* rs4825476 (*GRIA3*)) [31]. These results broaden the perspective of an explanation for both diseases’ etiopathophysiology, indicating a biological interaction between glutamate (*GRIA3*) and serotonin (*TPH2, SLC18A*1) pathways or factors associated with neurogenesis (*CDH9, NTRK3, OLIG2*) [31]. The epidemiologic data concerning the incidence of obsessive-compulsive symptoms in individuals suffering from AN, with the distinction between these subtypes, remains ambiguous. Some sources emphasize the fact that these symptoms are more prevalent among the bulimic type group [1, 48], while others describe them as particularly characteristic of the population with the restrictive type of AN [18].

Apart from the urge to maintain cleanliness and a slim outline or to make excessive efforts to perform all tasks immaculately, AN sufferers also exhibit specific obsessions and/or compulsions which may affect the progression of the condition [13, 24]. These include persistent thoughts related to food, weight loss and body self-perception, obsessive meal planning, meticulous calculation of each meals calorific value or to other compulsive behaviors, such as repeated body weight measurements (body checking behaviors), habitual compensating behaviors directed at the prevention of body mass increase (e.g., strenuous exercises or induction of vomiting) [14, 19]. For AN patients numerous food-related rituals are very important (e.g., arranging the cutlery in a particular way or eating certain foods in a specific order) [13]. Obsessive thoughts and/or compulsive behaviors in people suffering from AN can be classified as other factors influencing the permanence of abnormal dietary habits mainly due to difficulty in controlling and preventing obsessions and compulsions [3, 10, 61].

## Conclusion

The deviated attitude towards food of AN patients is conditioned by a number of complex factors. With regard to somatic causes, a specific activity of the neurotransmitters which regulate the mood of affected individuals in such a way that the process of eating causes anxiety, and restricting the diet induces a better mood, may be distinguished. Disturbances in ghrelin secretion, and the relevant response, are also important factors influencing food intake. The treatment of AN patients should also involve affective—motivational aspects. It is crucial to bear in mind the importance of the patient’s overestimation of meal portion sizes and the lack of nutritional knowledge which justify the need for nutritional re-education during treatment. Obsessive counting of a meal’s calorific value, ritualized behaviors related to food consumption, multiple checking of body mass and measurements, as well as a number of other compensating behaviors which may also develop into compulsions, further hinder the attainment of progress in therapy.

The vast diversityof restrictive dietary styles manifested by AN patients presented above confirm the complex nature of anorexia nervosa, a disorder often ignored by public opinion and perceived merely as a dietary caprice. Attempts aimed at understanding the operating mechanisms influencing the adherence to abnormal dietary attitudes may contribute to greater effectiveness of therapy and to diminishing the risk of disorder recurrence.
